# The Relationship between Crystalline Lens Power and Refractive Error in Older Chinese Adults: The Shanghai Eye Study

**DOI:** 10.1371/journal.pone.0170030

**Published:** 2017-01-23

**Authors:** Jiangnan He, Lina Lu, Xiangui He, Xian Xu, Xuan Du, Bo Zhang, Huijuan Zhao, Jida Sha, Jianfeng Zhu, Haidong Zou, Xun Xu

**Affiliations:** 1 Department of Preventative Ophthalmology, Shanghai Eye Disease Prevention and Treatment Center, Shanghai, China; 2 Department of Ophthalmology, Shanghai General Hospital, Shanghai Jiao Tong University, Shanghai, China; 3 Center of Disease Control and Prevention of Baoshan District, Shanghai, China; 4 Center of Disease Control and Prevention of Xuhui District, Shanghai, China; National Eye Institute, UNITED STATES

## Abstract

**Purpose:**

To report calculated crystalline lens power and describe the distribution of ocular biometry and its association with refractive error in older Chinese adults.

**Methods:**

Random clustering sampling was used to identify adults aged 50 years and above in Xuhui and Baoshan districts of Shanghai. Refraction was determined by subjective refraction that achieved the best corrected vision based on monocular measurement. Ocular biometry was measured by IOL Master. The crystalline lens power of right eyes was calculated using modified Bennett-Rabbetts formula.

**Results:**

We analyzed 6099 normal phakic right eyes. The mean crystalline lens power was 20.34 ± 2.24D (range: 13.40–36.08). Lens power, spherical equivalent, and anterior chamber depth changed linearly with age; however, axial length, corneal power and AL/CR ratio did not vary with age. The overall prevalence of hyperopia, myopia, and high myopia was 48.48% (95% CI: 47.23%–49.74%), 22.82% (95% CI: 21.77%–23.88%), and 4.57% (95% CI: 4.05–5.10), respectively. The prevalence of hyperopia increased linearly with age while lens power decreased with age. In multivariate models, refractive error was strongly correlated with axial length, lens power, corneal power, and anterior chamber depth; refractive error was slightly correlated with best corrected visual acuity, age and sex.

**Conclusion:**

Lens power, hyperopia, and spherical equivalent changed linearly with age; Moreover, the continuous loss of lens power produced hyperopic shifts in refraction in subjects aged more than 50 years.

## Introduction

Lens power plays an important role in the development of refractive error and usually changes along lifespan[1]. In children, lens power decreases with age; lens power decreases remarkably in infants who are one to two years old [2–4]. In growing children, lens power decreases due to the development of myopia [5, 6]. In adulthood, hyperopia increases with aging; this problem arises due to a reduction in lens power in aged population [7]. In later stages of life, cataract develops in some individuals. In such cases, there is a myopic shift in the ocular refractive error, which is attributed to an increase in the refractive power of lens[8]. Although lens power changes throughout life, it undergoes significant changes in both childhood and adulthood. However, we find it surprising to know that a few epidemiological studies about this topic, especially in older subjects [8, 9].

As the lens power cannot be measured in vivo and calculation of the power of the lens inside the eye is not straightforward[10], but can be calculated based on the other ocular components. Bennett and Rabbetts presented an in vivo formula to determine the power of lens [11]. Based on this formula, we can calculate lens power by considering distance refraction, corneal power, anterior chamber depth, and axial length. In this formula, certain constants have been revised recently. Furthermore, this improved formula can be used for calculating the mean values of crystalline lens power in case of studies performed using IOL Master biometry when the lens thickness is not known [11, 12]. The accuracy of such formulas depends on the validity of measurements of biometric parameters, which are included in the formula. Such formulas ensure the realization of large-scale epidemiology research studies investigating lens power.

Currently, several studies have reported about the various aspects of ocular biometry, particularly axial length and corneal power [13–19]. However, there are no scientifically robust studies to elucidate the distribution and impact of lens power in an adult Chinese population, which play a pivotal role in the refractive adjustment of the eye. Therefore, we conducted a population-based study to determine the lens power in an adult Chinese population, living in the suburban and urban areas of Shanghai in eastern China.

## Materials and Methods

### Sampling and Enumeration

This study was approved by the Ethics Committee of Shanghai General People Hospital, Shanghai, China. The study was conducted in accordance with the tenets of the World Medical Association’s Declaration of Helsinki. This study is a population-based, cross sectional study that was conducted on adults who were at least 50 years old. We examined the subjects from August 2014 to November 2014 in Shanghai. We included subjects living in the Baoshan and Xuhui districts of Shanghai, which represented the suburban and urban regions of Shanghai with moderate socioeconomic profile. The sampling frame was constructed using geographically defined clusters, which were based on the data of registered residents. The clusters’ boundaries were defined that each cluster contained a population of approximately 1000 individuals (all ages). Depending on the percentage of population that was older than 50 years of age, we randomly selected 24 and 19 clusters (with equal probability) from the sampling frame of Baoshan and Xuhui districts of Shanghai [20].

The inclusion criteria for subjects were as follows: the subjects were at least 50 years old; they were residents of the selected study districts for more than six months. Using the Household Resident Register Record administrated by the district administration, we identified households with eligible subjects. Then, we collected the address, name of household head, name of subjects, and date of birth. In total, we identified 6769 eligible subjects from this register. Then, we visited these households individually to verify that the enumerated subjects still resided at these addresses. After meeting these subjects, we arranged appointments for examining their eyes. After explaining the purpose of the study to these enumerated subjects, we also mentioned the risks and benefits of the examination. Thereafter, we obtained a written, informed consent letter from these subjects. We excluded eyes with iatrogenic or pathologic conditions, because these conditions compromised the measurement of refraction. Thus, we excluded individuals who had undergone a cataract surgery previously. We also excluded eyes that had any of the following issues: cataract, media opacities, significant retinal problems, and optic atrophy.

### Eye Examination

We set up an examination site for the community of included subjects; most subjects could reach the examination site by walking for half an hour. The identity of subjects was verified using the official photo identity cards of subjects. A trained interviewer provided a standard questionnaire to these subjects. Thus, we collected the details of their ophthalmic history and their educational background. An optometrist used an autorefractor (Topcon KR 8900; Topcon Corporation, Tokyo, Japan) to measure the non-cycloplegic refraction in these subjects. Distance visual acuity was measured using a log-MAR (logarithm of the minimum angle of resolution) tumbling E chart (Precision Vision, Villa Park, IL, USA) and a standard illumination box, which was kept at a distance of 4 m from these subjects. We recorded the presenting visual acuity with habitual correction. In subjects having a presenting visual acuity of less than 20/40 in either eye, we determined the best corrected vision using the results of auto-refraction. Thus, we implemented the necessary subjective refinement in these individuals with impaired vision. We measured the anterior chamber depth (ACD), corneal power (CP), and axial length (AL) of the globe using IOL master (IOL-Master; version 5.02, Carl Zeiss Meditec, Oberkochen, Germany).

### Calculation of Lens Power and Definitions

The lens power was calculated using the modified Bennett and Rabbetts formula:

$$P_{L}=\frac{L(S_{CV}+K)-1000\text{n}}{\left(L\text{-}\;ACD\text{-}\;C_{BR})(\frac{ACD+C_{BR}}{1000\text{n}}(S_{CV}+K)\;\text{-}\;1\right)}$$ (1)

with L the axial length, S_*CV*_ = SE/(1–0.014 SE) the spherical equivalent refraction at the corneal vertex, K the corneal power, n = 1.336 the refractive index of aqueous and vitreous humors, ACD the anterior-chamber depth, and C_*BR*_ = 2.564 mm the average distance between thin lens position and anterior lens surface. [11]

Since the refractive lens power and spherical equivalent of the left and right eyes were similar (r = 0.48, *P* < 0.001; r = 0.84, *P* < 0.001), the right eyes were chosen to represent a specific individual. The refraction data were converted to spherical equivalence (SE: sphere +1 ⁄ 2 cylinder). Myopia was defined as SE < –0.5 D; high myopia was further defined as SE < –6D. Hyperopia was defined as SE >0.5 D, and astigmatism was defined as cylinder power >0.75D. Furthermore, the axial length/corneal radius (AL/CR) ratio was defined as the axial length divided by the mean corneal radius of curvature. Education level was divided into 6 grades: 6 = higher than master's degree; 5 = bachelor's degree; 4 = high school education (grades 9–12); 3 = junior high school (grades 6–9); 2 = primary school (grades 1–6); 1 = less than primary school.

### Statistical Analysis

The linear regression model was used to investigate the independent effects of ocular biometry and other demographic factors on lens power and spherical equivalents. For constructing stepwise multivariate models, we included potential risk factors in multivariate regression models (α_in_ = 0.15; α_out_ = 0.15). We also performed stepwise multiple linear regression analysis using collinearity diagnostics to determine crystalline lens power and spherical equivalents. Furthermore, we identified the ocular components that were significantly correlated with lens power and spherical equivalents. All data analyses were performed on a computer equipped with statistical software (Statistical Analysis System, ver. 9.3; The SAS Institute, Cary, NC, USA). The graphs were also created on a computer with statistical software (SPSS statistical software, ver. 19.0; SPSS Inc., Chicago, IL, USA).

## Results

We examined 6769 subjects who were more than 50 years old. Refraction data were considered inappropriate for analysis and thus were excluded in 670 right eyes, which included aphakia/pseudophakia (59 eyes), significant cataract that led to best corrected VA <20/40(532 eyes), corneal opacity and scar (5 eyes), optic atrophy (8 eyes), retinal disease (46 eyes), glaucoma with significant postoperative complication and vision loss (18 eyes), poor cooperation due to mental retardation (2 eyes). After these exclusions, data of 6099 eyes was available for analysis. The mean age of these subjects was 62.56 ± 8.00 years (range: 50–96), and 3545 subjects (58.14%) were women. The mean ages of men (Mean±SD:63.61±8.15) were significantly greater than those of women (Mean±SD:61.80±7.80) (t-test = 8.79, *P* < 0.0001).

**Fig 1:**
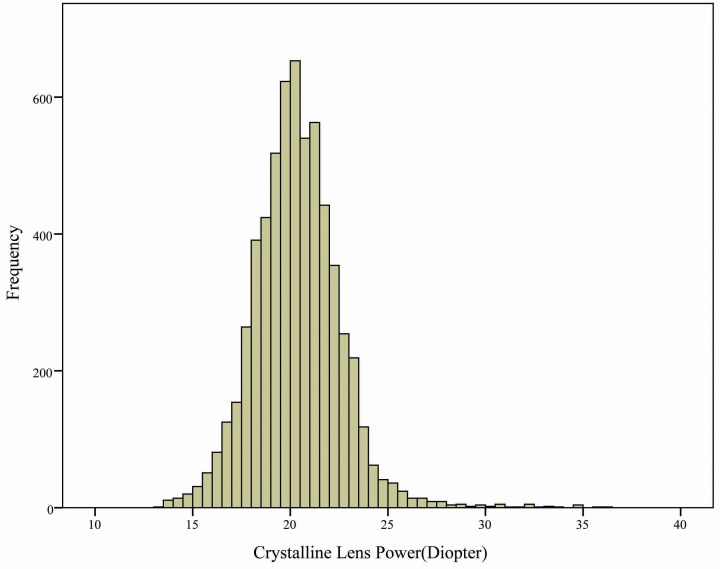
Histogram showing distribution of refractive lens power in Shanghai eye study 2014.

**Fig 2:**
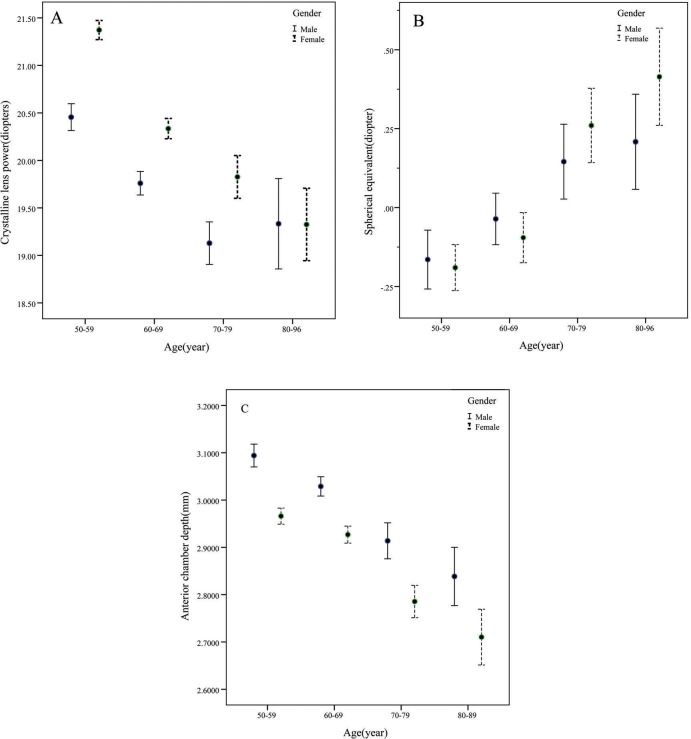
Mean lens power, spherical equivalent and anterior chamber depth partitioned by age and sex group. error bars represent 95% confidence intervals.

Table 1 presents the mean, standard deviation of lens Power, spherical equivalent, axial length, anterior chamber depth, corneal power, AL/CR ratio of individuals included in this study; the data is stratified according to gender and various age groups. The mean refractive lens power was 20.34 ± 2.24 D (range: 13.40–36.08) (Fig 1). In general, lens power decreased with age in an approximately linear fashion; however, lens power was consistently higher in women at an average of 0.81D (Fig 2A). The spherical equivalence increased with age in an approximately linear fashion; however, there was no difference between the spherical equivalence of men and women (Fig 2B). The axial length did not change with age and gender; however, it was consistently shorter in women at an average of 0.42 mm. The mean anterior chamber depth decreased with age in an approximately linear fashion; compared with men, it was shallower in women at an average of 0.1 mm (Fig 2C). Corneal power did not change with age, but it was consistently greater in women, with a mean sex difference of 0.58 D. AL/CR ratio did not change with age but it was lower in women, with a mean sex difference of 0.01.

**Table 1:** Distribution of Ocular Biometry in Chinese Adults.

Age Group	N	Lens Power(D)	SE(D)	AL(mm)	ACD(mm)	CP (D)	AL/CR ratio
Men							
50–59	861	20.46±2.11	-0.33±2.79	23.75±1.41	3.09±0.36	43.97±1.38	3.09±0.17
60–69	1159	19.76±2.15	-0.07±2.84	23.82±1.25	3.03±0.35	43.86±1.41	3.09±0.16
70–79	398	19.13±2.27	0.29±2.41	23.77±1.19	2.91±0.39	43.90±1.37	3.09±0.15
80–96	135	19.33±2.80	0.40±1.72	23.69±0.92	2.84±0.36	43.78±1.52	3.07±0.11
All	2553	19.87±2.24	-0.08±2.72	23.78±1.28	3.02±0.37	43.90±1.40	3.09±0.16
Women							
50–59	1528	21.37±2.00	-0.38±2.91	23.31±1.35	2.97±0.34	44.45±1.44	3.07±0.17
60–69	1462	20.33±2.08	-0.19±3.10	23.46±1.43	2.93±0.35	44.48±1.45	3.09±0.18
70–79	423	19.83±2.35	0.52±2.47	23.21±1.14	2.79±0.36	44.60±1.38	3.07±0.15
80–96	132	19.33±2.21	0.83±1.79	23.24±0.88	2.71±0.34	44.39±1.45	3.06±0.10
All	3545	20.68±2.18	-0.15±2.93	23.36±1.35	2.92±0.35	44.48±1.44	3.08±0.17
Men and Women							
50–59	2389	21.04±2.09	-0.36±2.87	23.47±1.39	3.01±0.35	44.28±1.44	3.08±0.17
60–69	2621	20.08±2.13	-0.14±2.99	23.62±1.37	2.97±0.36	44.20±1.47	3.09±0.17
70–79	822	19.49±2.34	0.41±2.44	23.48±1.20	2.85±0.38	44.26±1.42	3.08±0.15
80–96	267	19.33±2.52	0.61±1.76	23.47±0.92	2.78±0.36	44.08±1.51	3.06±0.11
All	6099	20.34±2.24	-0.12±2.84	23.53±1.34	2.96±0.36	44.23±1.45	3.08±0.17
Adjusted* β(Age)		-0.67	0.04	-0.005	-0.08	-0.006	-0.0009
P(Age)		<0.0001	<0.0001	0.82	<0.0001	0.7832	0.7426
Adjusted* β(Sex)		0.7	0.07	-0.43	-0.12	0.58	-0.02
P(Sex)		<0.0001	0.81	<0.0001	<0.0001	<0.0001	0.0004

* Adjusted regression coefficient suggested by multiple linear regression model where age and sex were included as independent variables.

In univariate analysis, refractive lens power was significantly associated with the following parameters: age (β(95%CI) = –0.08(–0.09,–0.07),*P* < 0.0001), sex(β(95%CI) = 0.81(0.70,0.92),*P* < 0.0001), refractive error (β(95%CI) = –0.03(-0.05,-0.01),*P* = 0.005), axial length (β(95%CI) = –0.70 (–0.74,–0.66),*P* < 0.0001), ACD(β(95%CI) = –1.03(–1.18, –0.87),*P* < 0.0001), corneal power(β(95%CI) = 0.07(0.04,0.11),*P* = 0.001), AL/CR ratio (β(95%CI) = –5.90(–6.21,–5.60),*P* = 0.0002), region(β(95%CI) = –0.57(–0.68,–0.46),*P* < 0.0001), education level(β(95%CI) = –0.08(–0.14,–0.03),*P* = 0.002), and best corrected visual acuity(β(95%CI) = –1.11(–1.59,–0.63),*P* < 0.0001). The correlation between lens power and ocular biometry was adjusted for age and gender. After making adjustments for age and gender, refractive lens power was not significantly associated with refractive error (β(95%CI) = –0.01(-0.02,0.01),*P* = 0.54),CP (β(95%CI) = 0.03(–0.01,0.06),P = 0.16) and BCVA (β(95%CI) = –0.17(–0.63,0.30),P = 0.49).

In stepwise multivariate analysis, we defined lens power as the dependent variable. Other parameters, such as age, sex, refractive error, axial length, corneal power, ACD, suburban/urban region, education level, and best corrected visual acuity were defined as independent variables. In this analysis, we found that lens refractive power was significantly associated with the following parameters: age(β(95%CI) = –0.00(–0.01,–0.00),*P* < 0.0001), sex(β(95%CI) = 0.04(0.02,0.07),*P* = 0.0002), refractive error (β(95%CI) = –1.45(–1.46,–1.44),*P* < 0.0001), axial length(β(95%CI) = –3.96(–3.98,–3.93),*P* < 0.0001), ACD(β(95%CI) = 1.84(1.80,1.88),*P* < 0.0001), corneal power(β(95%CI) = –1.44(–1.45,–1.43),*P* < 0.0001), region(β(95%CI) = –0.02(–0.04,0.00),*P* = 0.13), and best corrected visual acuity (β(95%CI) = –0.20(–0.30,–0.10),*P* <0.0001)(Table 2).

**Table 2:** Multivariate analysis of the association between lens Power and ocular biometric parameters in participants.

Parameters	Nonstandardized Regression Coefficient (95% CI)	Standardized RegressionCoefficient	Variance Inflation Factor	P value
Age(y)	-0.00(-0.01,-0.00)	-0.01	1.24	<0.0001
Sex	0.04(0.02,0.07)	0.01	1.10	0.0002
SE(D)	-1.45(-1.46,-1.44)	-1.84	5.02	<0.0001
ACD(mm)	1.84(1.80,1.88)	0.30	1.59	<0.0001
AL(mm)	-3.96(-3.98,-3.93)	-2.36	6.92	<0.0001
CP(D)	-1.44(-1.45,-1.43)	-0.93	2.14	<0.0001
Suburban/Urban Region	-0.02(-0.04,0.00)	-0.00	1.06	0.13
BCVA(Log MAR)	-0.20(-0.30,-0.10)	-0.01	1.17	0.0004

Log MAR = logarithmic value of the minimal angle of resolution.

The mean refractive error was -0.12±2.84D (range: –26.25 to 10.38) (Fig 2). In Table 3, we have summarized the prevalence of myopia, high myopia, hyperopia, and astigmatism according to gender and age groups.The prevalence of overall hyperopia (SE > 0.5 D), myopia (SE <- 0.5 D), high myopia (SE<–6 D), and astigmatism (cylinder > 0.75 D) were 48.48% (95% confidence interval CI: 47.23–49.74), 22.82%(95% CI, 21.77–23.88), 4.57% (95% CI: 4.05–5.10), and 49.29%(95% CI:48.03–50.54), respectively. The χ^2^ trend test suggests that the prevalence of hyperopia, myopia, high myopia, and astigmatism was associated with age, whereas any refractive error was not dependent on the sex of the individual (Table 3).

**Table 3:** Distribution of Ocular Biometry in Chinese Adults.

Age Group	N	Hyperopia(>+0.5 D)	Myopia(< -0.5 D)	High Myopia(< -6.0 D)	Astigmatism(>0.75 D)
Men					
50–59	861	42.28(38.98–45.58)	23.23(20.41–26.05)	5.23(3.74–6.71)	38.68(35.42–41.93)
60–69	1159	51.86(48.98–54.73)	23.21(20.78–25.64)	3.88(2.77–4.99)	47.71(44.84–50.59)
70–79	398	54.77(49.88–59.66)	22.61(18.50–26.72)	2.26(0.80–3.72)	64.07(59.36–68.78)
80–96	135	52.59(44.17–61.02)	17.04(10.70–23.38)	0.74(-0.71–2.19)	68.15(60.29–76.01)
All	2553	49.12(47.18–51.06)	22.80(21.17–24.42)	3.92(3.16–4.67)	48.30(46.36–50.23)
Women					
50–59	1528	40.64(38.18–43.10)	24.48(22.32–26.63)	5.04(3.94–6.14)	41.36(38.89–43.83)
60–69	1462	50.75(48.19–53.32)	23.26(21.09–25.42)	6.16(4.92–7.39)	53.83(51.27–56.39)
70–79	423	61.23(56.59–65.87)	17.97(14.31–21.63)	2.60(1.08–4.12)	63.36(58.77–67.95)
80–96	132	61.36(53.06–69.67)	15.15(9.03–21.27)	0.76(-0.72–2.24)	65.15(57.02–73.28)
All	3545	48.04(46.39–49.68)	22.85(21.47–24.23)	5.05(4.33–5.77)	50.01(48.37–51.66)
Men and Women					
50–59	2389	41.23(39.26–43.20)	24.03(22.31–25.74)	5.11(4.22–5.99)	40.39(38.43–42.36)
60–69	2621	51.24(49.33–53.15)	23.24(21.62–24.85)	5.15(4.30–6.00)	51.13(49.21–53.04)
70–79	822	58.03(54.66–61.40)	20.19(17.45–22.94)	2.43(1.38–3.49)	63.63(60.34–66.91)
80–96	267	56.93(50.99–62.87)	16.10(11.70–20.51)	0.75(-0.29–1.78)	66.67(61.01–72.32)
All	6099	48.48(47.23–49.74)	22.82(21.77–23.88)	4.57(4.05–5.10)	49.29(48.03–50.54)
*X*^*2*^		9.2712	-3.2073	-3.9339	13.2113
P (age)*		<0.0001	0.0013	< .0001	< .0001
*X*^*2*^		0.7096	0.0018	5.5730	1.7241
P (sex)*		0.3996	0.9664	0.0182	0.1892

*Adjusted regression coefficient suggested by multiple linear regression model where age and sex were included as independent variables.

In univariate analysis, the refractive error was significantly associated with the following parameters: age (β(95%CI) = 0.04(0.03,0.04),*P* < 0.0001), refractive lens power (β(95%CI) = –0.05(–0.08,–0.01),*P* = 0.0045), axial length(β(95%CI) = –1.67(–1.70, –1.64),*P* < 0.0001), ACD(β(95%CI) = –2.88(–3.06,–2.69),*P* < 0.0001), corneal power (β(95%CI) = –0.10(–0.15,–0.05),*P* < 0.0001), AL/CR ratio (β(95%CI) = –14.77(–14.99,–14.55),*P* < 0.0001), region(β(95%CI) = –0.65(–0.80,–0.51),*P* < 0.0001), education level (β(95%CI) = –0.39(–0.45,–0.32),*P* < 0.0001), and best corrected visual acuity (β(95%CI) = –0.66(–0.72,–0.61),*P* < 0.0001). Refractive error was not significantly related with sex (β (95%CI) = –0.07(–0.22, 0.07), *P* = 0.3241). To determine the correlation between refractive error and ocular biometry, we made adjustments in the age and gender of subjects (Table 4).

**Table 4:** Linear Regression Model for the Determinants of Refractive Error (Univariate Analysis).

Parameters	Adjusted Non-standardized Regression Coefficient (95% CI)	Standardized Regression Coefficient	P value
Lens power	-0.01(-0.04,0.02)	-0.01	0.53
AL	-1.71(-1.75,-1.68)	-0.81	<0.0001
ACD	-2.91(-3.09,-2.72)	-0.37	<0.0001
CP	-0.10(-0.15,-0.05)	-0.05	<0.0001
AL/CR	-14.82(-15.03,-14.61)	-0.87	<0.0001
Suburban/Urban Region	-0.76(-0.90,-0.62)	-0.13	<0.0001
Level of education	-0.36(-0.42,-0.29)	-0.14	<0.0001
BCVA(LogMAR)	-0.74(-0.79,-0.68)	-0.31	<0.0001

Adjusted for age and sex. Log MAR = logarithmic value of the minimal angle of resolution.

In stepwise multivariate analysis, we defined refractive error as the dependent variable. Other parameters, such as age, sex, refractive lens power, axial length, corneal power, ACD, suburban/urban region, educational background, and best corrected visual acuity were defined as independent variables. Refractive error was significantly associated with age (β(95%CI) = –0.01(-0.02,0.00),*P* = 0.1426), sex(β(95%CI) = 0.01(–0.00,0.03),*P* = 0.0558),refractive lens power(β(95%CI) = –0.66(–0.66,–0.66),*P* < 0.0001), axial length (β(95%CI) = –2.71(–2.71,–2.70),*P* < 0.0001), ACD(β(95%CI) = 1.26(1.24,1.29),*P* < 0.0001), corneal power(β(95%CI) = –0.99(–1.00, –0.98),*P* < 0.0001), and best corrected visual acuity (β(95%CI) = –0.02(–0.02,–0.01),*P* < 0.0001) (Table 5).

**Table 5:** Multivariate analysis of the association between refractive error and ocular biometric parameters in participants.

Parameters	Non-standardized Regression Coefficient (95% CI)	Standardized Regression Coefficient	Variance Inflation Factor	P value
Age(y)	-0.01(-0.02,0.00)	0	1.19	0.14
sex	0.01(-0.00,0.03)	0	1.09	0.06
Lens power(D)	-0.66(-0.66,-0.66)	-0.52	1.4	<0.0001
AL(mm)	-2.71(-2.71,-2.70)	-1.27	2.14	<0.0001
ACD(mm)	1.26(1.24,1.29)	0.16	1.48	<0.0001
CP(D)	-0.99(-1.00,-0.98)	-0.5	1.3	<0.0001
BCVA (LogMAR)	-0.02(-0.02,-0.01)	-0.01	1.15	<0.0001

logMAR = logarithmic value of the minimal angle of resolution.

## Discussion

This study is a population based cohort study. The surveys were carried out on a randomly selected sample of individuals living in suburban and urban areas of Shanghai. In total, we selected 42 basic sampling units, which represented the aged population of China. We will follow-up the changing trend of refractive lens power and other ocular biometry in people who are at least 50 years old. In this study, we only analyzed the refractive lens power and ocular parameters of normal eyes; we excluded individuals with eye diseases, such as cataract, corneal opacity, and fundus disease. Thus, we explored the association between refractive lens power and refractive error under normal physiological conditions. The basic cross-sectional data provides a good foundation for future follow-up research. This is the first large scale study to explore the relationship between refractive lens power and refractive error in older adults in china.

In this study, we found that the men had lower lens power, longer eyes, flatter corneas, deeper anterior chambers, and higher AL/CR ratio than women of all ages. However, the distributions of refractive errors were similar in both men and women. Lens power and anterior chambers depth decreased with age, while refractive error increased with age. However, there was no significant difference in AL, CP, AL/CR ratio of subjects belonging to different age groups.

**Fig 3:**
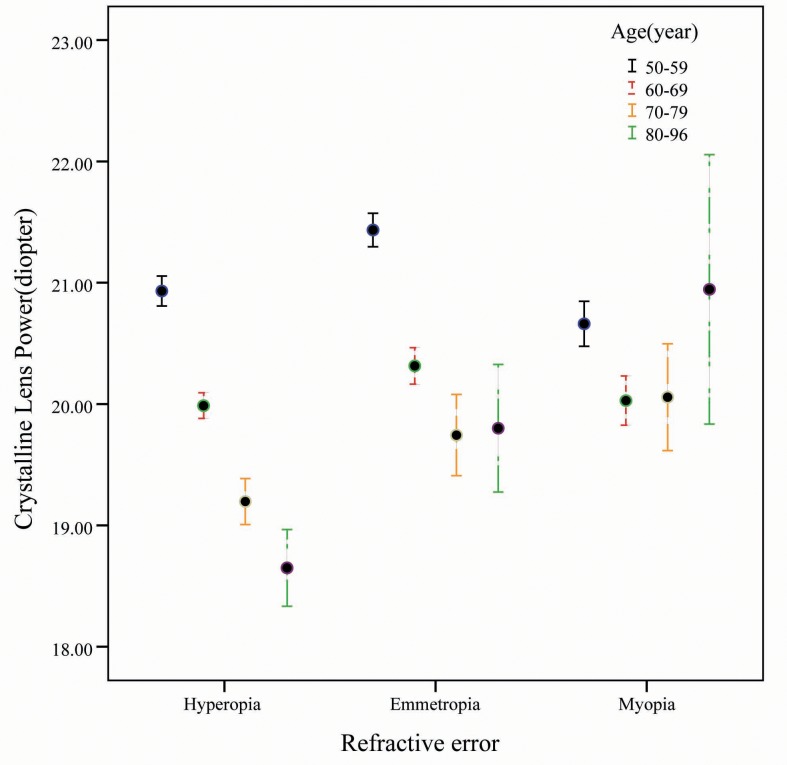
Mean crystalline lens power partitioned by refractive error type and age group. error bars represent 95% confidence intervals.

In our study, the mean refractive lens power was 20.34 ± 2.24D, which was lower than that of eyes of people living in central India and Shahroud [1, 9]. This is because of different formula used to calculate the lens power, different ethnic group or the older age distribution. In stepwise multivariate regression analysis, we found that most determinants of lens power are associated with shorter axial length, higher spherical equivalence, and steeper corneal power; Additionally, most determinants of lens power are also associated to some extent with older age, BCVA, suburban region, and females. In our study we could not establish a linear relationship between lens power and refractive error. Furthermore, we found that the refractive lens power of emmetropia was the highest, while the refractive lens power of hyperopia was higher than that of myopia (Fig 3)[6, 8, 18, 21, 22]. This is in contrast to the positive correlation finding in children studies[6],with hypermetropes having higher refractive error than emmetropes or myopes. The positive correlation between lens power and refractive error in myopes accords with the relationship between lower lens power and increasing axial length in childhood. For myopic eyes, they are generally longer which need a weaker power lens to compensate, while hypermetropic eyes are usually shorter which require a stronger lens in childhood. In adulthood, some cases of emmetropia and lower myopes are driven to hyperopic condition with a reduction in lens power [1], so the group of hypermetropes will consist of a mixture of newly developed hypermetropes with lower lens power and a small portion of persistent hypermetropes with a higher lens power. This new balance could reverse the correlation between lens power and refractive error, and would explain why in the present study lens power is not linearly associated with refractive error.

The overall prevalence of hyperopia, myopia, and high myopia was 48.48% (95% CI: 47.23–49.74), 22.82% (95% CI: 21.77–23.88) and 6.03% (95% CI: 5.44–6.63), respectively. These findings were similar to those of a study conducted in Guangzhou province of China [14]. The prevalence of hyperopia and spherical equivalence in subjects of different aged group increased linearly with age; this result agreed with the findings of a previous study, which reported that there was a hyperopia shift with aging [14, 16]. This study excluded cataract patients. Furthermore, myopia shift factor caused by cataract was also removed; therefore, compared to previous studies, the degree of hyperopic shift was more obvious in this study [14, 16]. In stepwise multivariate analysis, we determined standardized correlation coefficient and found that refractive error is strongly correlated with axial length, lens power, corneal power, and anterior chamber depth; however, it is slightly correlated to BCVA, age, and sex. Because corneal power is basically stable after the first two years of life and axial length is generally to keep stable after 20 to 30 years old [1], the changes of refractive error may be mostly due to changes in lens power and ACD. In our study the overall prevalence of hyperopia, spherical equivalence, and refractive lens power varied with the age of subjects; however, axial length and corneal power did not vary with the age of subjects. Therefore, the changes in lens power and ACD may be associated with hyperopic shifts.

We need to elucidate the mechanisms bringing about changes in lens power, anterior chamber depth, and hyperopic shifts. The changes in gradient index have also been associated with hyperopic shifts, which lead to the development of hyperopic refractive errors in aging adults [7, 22–24]. The lens grows from the surface, sinking fibers in its deeper layers. But, the newly laid fibers have greater water content and lower refractive index than the older ones. Thus, a gradient of refractive index is produced, increasing from the cortex to the center of the lens[25]. The total power of the lens originates from its surface, and it is influenced by the curvatures of the lens. However, there exists an internal power that defines the gradient index of refraction in the lens. The profile of gradient index structure governs the differential power of the lens. In this profile, a low index is at the periphery, while a high index is at the center of the lens; however, this profile develops a central plateau in an ageing population [26, 27]. As the profile of the peripheral gradient becomes more abrupt, a plateau develops and the ACD becomes relevantly shallow. Therefore, the change in the gradient index and the profile lead to the loss of lens power in adults, causing a major hyperopia shift. This explanation is contrast to lens paradox [28–30] that the maintenance of emmetropia in the adult in spite of continuing lens growth with increasing lens thickness and increasing lens curvature.

We need to mention the potential limitations of our study. Firstly, any population based study’s major concern is non-participation of subjects. In this study, the response rate was 94.34%; however, differences between participants and non-participants could have led to a selection bias. Secondly, our study data was based on non-cycloplegic refractions in adults, so the accommodation of lens cannot be excluded. As a result, the data of this study is relatively less accurate. Thirdly, it may not be appropriate to compare the mean lens power of different studies, because the measurements and formula used in different studies are different.

In conclusion, in the suburban and urban population of Shanghai, lens power changed linearly with age. Moreover, the prevalence of hyperopia and spherical equivalence also increased with age. However, axial growth and corneal power did not change in subjects belonging to different age groups. Furthermore, the continuous loss of lens power caused hyperopic shifts in an adult population that included subjects who were more than 50 years old. In addition, the lens power of hyperopic and myopic eyes was lower than that of emmetropic eye, a finding that contrasted with the ocular conditions of children.

## Supporting Information

S1 File The ocular biometry data of in shanghai eye study, N = 6099. Variable names included DIS(1 = Baoshan, 2 = Xuhui),sample name, age(y), sex(1 = male,2 = female), present visual acuity, lens power(D),SE(D), CP(D), AL(mm), ACD(mm).
(CSV)
